# Evaluation of Medication Package Inserts in Ethiopia

**DOI:** 10.1155/2022/8299218

**Published:** 2022-01-19

**Authors:** Haftom Gebregergs Hailu, Mengistie Yirsaw Gobezie, Khalid Beshir Tuem, Hildana Tewolde Gebremichael, Solomon Ahmed Mohammed

**Affiliations:** ^1^Department of Pharmacology and Toxicology, School of Pharmacy, College of Health Sciences, Mekelle University, Mekelle, Ethiopia; ^2^Department of Pharmacy, College of Medicine and Health Sciences, Wollo University, Dessie, Ethiopia

## Abstract

**Background:**

Patients require accurate and reliable information to help them use their medications safely and effectively. Inadequate patient knowledge may contribute to medication nonadherence which could negatively affect treatment outcomes. The purpose of this study was to evaluate the presentation and completeness of medication package inserts (MPIs) which are available in the Ethiopian market.

**Methods:**

A cross-sectional document review was performed in February and March of 2019. All MPIs which were authorized by EFDA to sell in the Ethiopian market and available during the data collection period were considered.

**Results:**

The mean overall completeness score of 200 MPIs was 18.39 ± 4.30. Of the 200 MPIs, only 20% were from domestic pharmaceutical companies. Antimicrobials represented 24% of the total MPIs. Topical preparations, cardiovascular drugs, gastrointestinal drugs, and nonsteroidal anti-inflammatory drugs, accounted for 12.5%,12.5%, 11%, and 9% of the MPIs, respectively. The majority of the MPIs presented information about the drug's use during pregnancy and lactation, 77.0% and 74.0%, respectively. However, only half of the MPIs, 49.5%, gave information about special warnings and precautions. Only a few of the MPIs provided information about instructions to convert tablets or capsules into liquid forms and the possibility of tablet splitting, 4.8% and 8.7%, respectively. Furthermore, only 1.0% had local language translation.

**Conclusion:**

The MPIs available in Ethiopia provide inadequate information including about the safety of drug products and local language translation. Regulatory authorities should implement stringent regulations to ensure the provision of vital information which extends beyond checking the mere presence of an MPI. They should also act to the possible standardization of MPIs.

## 1. Introduction

Drug treatment is the most common intervention by healthcare providers [1]. Patients require accurate and reliable information to help them use their medications safely and effectively [1, 2]. However, inadequate information provided by healthcare providers is common because of heavy workloads, many patients unable to retain verbal information they have been told for a long time [3], and misunderstanding of verbal information [4]. Insufficiency of pharmacological knowledge and communication skill by health professionals might also contribute [5]. Furthermore, both physicians and pharmacists broadly vary in the frequency and types of information they give about drug products [6]. As a result, there is consensus among the medical community about the need for high-quality written information for patients about their medications [7]. Written information about drug products such as the medication package inserts (MPIs) is one of the sources that patients use to obtain information about their medication [8].

MPIs are leaflets that are packed with both over-the-counter and prescription medicines to provide specific information about their indication, administration, precautions during use, dose/dosage, potential side effects, and contraindications [5, 9]. MPIs, which are also called patient package inserts or prescription drug labels, are provided by the medicine's manufacturer based on regulatory guidelines [2, 4]. It is an important source of medication information to both healthcare providers and patients especially in developing countries where access to contemporary medical information is limited [3, 10].

Inadequate patient knowledge may contribute to medication nonadherence which could negatively affect treatment outcomes. MPIs improve patients' understanding and contentment which advances adherence to treatment [11]. In Ethiopia, nonadherence to treatment and poor knowledge about medications have been correlated by different studies [12–14]. However, an MPI with wrong or missed information may lead to negative patient responses and reactions [7]. Besides, incomplete and incorrect medication information may promote irrational drug use and may have serious consequences, including disability and death [5, 15]. As a result, MPIs should be regulated not to be promotional, false, or misleading [16].

Many countries force MPIs to be included in the medication package [7]. The European Union requires all medicines to be marketed within its member states and to be packed with an MPI as a legal requirement [17]. In Ethiopia, providing an MPI with medicine is a precondition for marketing by the Ethiopian Food and Drug Authority (EFDA) (formerly known as Food, Medicine, and Health Care Administration and Control Authority of Ethiopia (FMHACA)). EFDA also states that “the MPI should not be described or presented in a manner that is false, misleading, or deceptive or is likely to create an erroneous impression regarding its use in any respect, either pictorially or in words” [18].

Even though MPIs are vital to providing reliable information, patients usually do not find the information they are looking for [10]. An online literature search reveals that there is no prior study about the MPIs in Ethiopia. Therefore, the purpose of this study was to evaluate the presentation and completeness of MPIs which were available in the Ethiopian market.

## 2. Methods

### 2.1. Study Area and Period

This study was conducted in Ethiopia. MPIs were collected in February and March of 2019. Ethiopia is one of the most populous countries in Africa, and the demand for pharmaceutical products in the country is high. This study was conducted in Mekelle city, which is the capital city of the Tigray region, around 783 kilometers from Addis Ababa, the capital city of Ethiopia. Currently, there are 3 governmental general hospitals, 1 comprehensive specialized hospital, 35 pharmacies, 47 drug stores, and 3 drug vendors rendering services in the town. Only drug products that are authorized by the regulatory body are sold in the Ethiopian market. The pharmaceutical sector mainly relies on imports from foreign countries and as a result, the international market remains the main source of important products that are not yet manufactured nationally.

### 2.2. Study Design

A cross-sectional document review was performed. Each MPI was read thoroughly to decide whether it contained the required information or not.

### 2.3. Study Population and Inclusion and Exclusion Criteria

All MPIs which were authorized by EFDA to sell in the Ethiopian market and available during the data collection period were considered. All available MPI's at community and hospital pharmacies (3 governmental general hospitals, 1 comprehensive specialized hospital, 35 pharmacies, and 47 drug stores) in the town at the date of data collection were included in the study.

### 2.4. Data Collection, Data Management, and Analysis

The MPIs were collected for 2 weeks from the hospital and community pharmacies located in Mekelle. The MPIs were collected and analyzed by the authors from hospital and community pharmacies The authors approached the person in charge of hospital and community pharmacies and requested MPIs. Medicines were not purchased and all available MPIs were searched in all packages. Medicines that did not contain MPIs were excluded. MPIs were randomly collected from the community and hospital pharmacies of Ethiopia from January to February of 2019. Duplicates were checked and discarded. A total of 200 MPIs were included in this study. A data collection format was prepared to gather the information regarding the presence of required information on each of the collected MPIs.

MPIs were evaluated for parameters that were extracted from EFDA's guideline for product registration. The MPI's classification was performed according to EFDA's medicine classification [18]. The parameters included indications, formulation, country of origin, name and address of the manufacturer, active ingredient, drug dose, storage conditions, mechanism of action, directions for use, inactive ingredients, pharmacokinetic information, date of last revision, duration of treatment, container package description, shelf life, provision of full information on request, sources of information/reference, presence of local language translation, and the retail price of the drug. In addition to this, MPIs were evaluated for the presence of safety information such as adverse drug reactions, contraindications, drug-drug interactions, drug-food interactions, pregnancy considerations, lactation considerations, pediatric considerations, geriatric considerations, special warning and precautions, abuse/dependence property, effect on the ability to drive and operate machines, overdose, and management, maximum dose, missing dose, handling, and disposal.

Besides, MPIs of solid dosage forms were assessed if they contained information about the instructions to convert tablets or capsules into liquid forms, the possibility of crushing and mixing with food or beverages, and the possibility of tablet splitting. The list of European Union member states was taken from its official website [19].

A scored one was given when all required information on an item was present; otherwise, a score of zero was given. Each item had one score, and the total score (28) was calculated by adding the scores of all items for an individual MPI's. Data were fed to and analyzed using the Statistical Package for Social Sciences software, version 22. Frequencies and percentages of the MPIs conforming to the selected parameters under observation were determined.

## 3. Results

**Figure 1:**
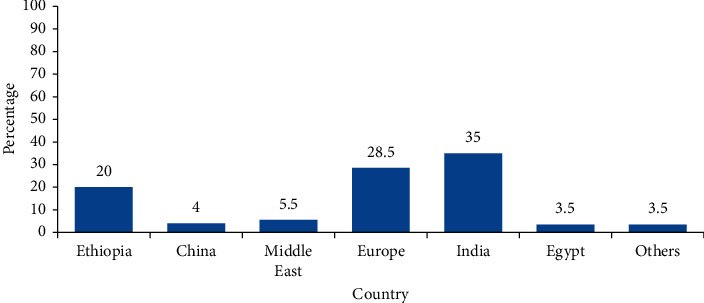
Classification of medication package inserts according to their country of origin (*n* = 200).

MPI's country of origin was classified into Ethiopia, India, China, Europe, the Middle East, Egypt, and others (Pakistan, Nigeria, Singapore, and Kenya). Middle Eastern countries included Qatar, United Arab Emirates, Bahrain, Saudi Arabia, Kuwait, Israel, Oman, Iran, Jordan and Lebanon, Syria, Yemen, and Iran. Of the 200 MPIs, only 20% were from domestic pharmaceutical companies. Drug products from India gained the highest proportion (35%), followed by Europe (28.5%). The Middle East, China, and Egypt accounted for 5.5%, 4%, and 3.5%, respectively (Figure 1). MPIs were analyzed for the type of dosage forms on which they were packed with and that shows 73.0% solid, 12.0% cream, 11.0% liquid, 3.0% injection, and 1.0% powder dosage forms.

**Figure 2:**
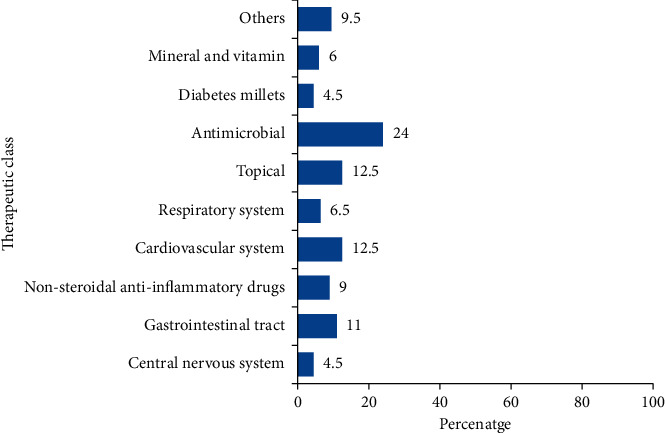
Classification of medication package inserts based on the therapeutic class of the drugs (*n* = 200).

The MPIs were also categorized based on their therapeutic class. Antimicrobials represent 24% of the total MPIs. Topical preparations, cardiovascular drugs, gastrointestinal drugs, nonsteroidal anti-inflammatory drugs, respiratory system drugs, and minerals and vitamins accounted for 12.5%, 12.5%, 11%, 9%, 6.5%, and 6% of the MPIs, respectively (Figure 2).

All of the MPIs provided information about the therapeutic class, indication, and country of origin (Table 1). From the total MPIs (200) evaluated, only 146 inserts were modifying solid dosage forms. Few of the MPIs have also included information about instructions to convert tablets or capsules into liquid forms, the possibility of crushing and mixing with food or beverages, and the possibility of tablet splitting, 4.8%, 4.1%, and 8.7%, respectively (Table 2).

MPIs were also analyzed for the presence of important safety information about drug products (Table 3). Nearly all of the MPIs presented information regarding the adverse reactions and contraindications, 95.5% and 94.5%, respectively. The majority of the MPIs, 80%, also inscribed about potential drug-drug interactions even though only 21% of them mentioned potential drug-food interactions. The mean overall completeness score of 200 MPIs was 18.39 ± 4.30 (range 7–28 variables).

## 4. Discussion

The Ethiopian rational drug use directive published in 2019 (‘Amharic' version, the official language of the EFDA along with English) states that patients should receive oral and written information about their medication's indication, dose/dosage, direction for use, precautions, contraindications, storage conditions, and side effects [20]. Physicians and pharmacists are expected to provide medication information. However, Ethiopian physicians are stressed with work overload, and the density of physicians to population ratio is very low [21, 22]. The practice of counseling during medication dispensary by Ethiopian pharmacists has also been reported to be unsatisfactory [23–25] (in 2015). Therefore, the need for quality MPIs is indispensable.

This study showed that most of the collected MPIs were for antimicrobials (24%). This might be attributed to three reasons. The first one is that communicable disorders are the leading cause of death in Ethiopia according to a 2015s report by the global burden of disease study [26]. The second explanation goes to the culture of inappropriate use of antimicrobials which has been reported in different parts of Ethiopia which might increase their demand [27–31]. The last justification is because self-medication practices even with prescription-only medications including antimicrobials are common phenomena in Ethiopia [32–34]. Moreover, the higher availability of antimicrobial MPI's might be the manufacture attempt to reduce the spread of resistance.

EFDA's guideline for registration of medicines states that the drug product summary characteristics to be submitted to the authority for registration should include the following information: the name of the product, strength, pharmaceutical form, indications, method of administration, contraindications, special warnings, and precautions, drug-drug, and other forms of interactions, drug use during pregnancy and lactation, effects on the ability to drive and use machines, undesirable effects, overdose, pharmacodynamic and pharmacokinetic properties, list of all excipients, incompatibilities with other pharmaceutical products, shelf life, special precautions for storage, nature, and contents of the container, instructions for use and handling and disposal, and last date of revision of the text. However, this same document does not specify which of the above information must be included in the MPI and to what extent. It simply demands the contents of the MPI to be in line with the submitted product summary characteristics [18]. This study revealed that the majority of the medications were imported from foreign countries: India, Europe, and the Middle East countries contributing 35%, 28.5%, and 5.5% of the total MPIs, respectively (Figure 1). MPIs from different countries might provide different information about a single drug product which might lead to unstandardized information overload.

Local language translation was not depicted in 98.5% of the MPIs. Contents were written in the English language except in two of the MPIs which provided Amharic translation. EFDA demands that drug product summary characteristics including MPIs required for registration to be presented in English [18]. This might be to mean that foreign languages other than English are not welcomed by the authority. However, even though the use of the English language in Ethiopia together with local and/or federal working languages is increasing, and its use is still limited to a small minority of educated economic and/or political elite [35, 36]. Furthermore, the role of English in Ethiopia is not even clearly mentioned either in the federal constitution or in the regional ones [35]. Ethiopian language is home to more than 75 languages with Amharic, Oromifa, and Tigrinya being the most commonly spoken languages [37]. Amharic is the working language of the federal government of Ethiopia [38]. As a result, English-only MPIs are not the best way to deliver medical information to the Ethiopian public.

All of the evaluated MPIs wrote the indication of the drug products they presented. However, directions for use of the products were not provided in 37.5% of the MPIs though it gives people the knowledge of how to use their medications appropriately. The duration of use was also not mentioned in 66.0% of the MPIs and they did not specify physician consultation. The maximum dose of medication and what to do when a dose is missed were also depicted only in 27.0% and 26.0% of the total MPIs. This happened although drug-related problems including unnecessary drug therapy, noncompliance, and too high doses have been reported in different parts of Ethiopia [28, 39, 40].

Nearly all of the MPIs provided information about the adverse effects and contraindications of the drug products, 95.5% and 94.5%, respectively. However, some important safety information was overlooked (Table 3). Information about drug-food interactions was provided only by 31.0% of the MPIs though diet can alter the pharmacokinetics and/or pharmacodynamics properties of drugs which might lead to toxicity or treatment failure [41]. Similarly, inactive ingredients were not listed in 47.0% of the MPIs. Intolerances and allergic reactions attributed to inactive ingredients have been reported [42]. It was also observed that the shelf life was skipped in 84.0% of the MPIs. If a medication is not used within its shelf life, both efficacy and safety might be compromised [43]. Handling and disposal were missed in 77.5% of the MPIs. Inappropriately disposed drugs might be harmful to humans, animals, and the environment [44].

The possibility of tablet splitting was provided only in 8.7% of the MPIs presented with tablet dosage forms. It is required when there is a need to split the medication into equal half so that patients can get the needed effectiveness from both halves of the tablet. However, not all tablet formulations are convenient for splitting. Splitting of extended-release preparations might cause an immediate release of the active ingredient which might lead to toxicity. In addition to this, splitting enteric-coated tablets might render them inactive by exposing them to gastric contents [45]. Similarly, the possibility of crushing and mixing with food or beverages and instructions to convert tablets or capsules into liquid forms was also depicted only in 4.1% of the MPIs. However, the possibility of tablet splitting, crushing, or mixing of tablets and capsules with food or beverages can cause a change in the rate of absorption of the preparations which can lead to a change in the plasma concentrations and consequently a change in the efficacy and safety of the drug products [46, 47].

The date of the last revision was not provided for 60.5% of MPIs which was higher when compared to a study conducted on south India which was found to be only 41.53% [16]. This shows that there is no way of knowing if the information provided in the leaflets is recent or outdated. The main reason could be that they were initially published years ago and are being used till now. Sources of information/reference were not provided in 98.0% of the MPIs which could have been used to identify where the information put on the MPIs came from. This might help to verify the validity of the presented information. The provision of full request information was not highlighted in the majority of the MPIs, 86.0%. As a result, access to detailed information on that specific drug will not be available for patients and health professionals easily.

## 5. Conclusion

Based on this study, we concluded that the MPIs available in Ethiopia provide inadequate information about the safety of drug products and local language translation. Drug companies should act responsibly and provide information in their MPI's including an additional insert with the local language(s). Regulatory authorities should implement stringent regulations to ensure the provision of vital information which extends beyond checking the mere presence of an MPI. They should also act to the possible standardization of MPIs.

Moreover, it should be better to research assess the availability of MPIs and drug usage. The actual correlations might be proven by drug utilization studies.

## Figures and Tables

**Table 1:** Presence of required information in medication package inserts (*n* = 200).

Variables	Frequency	Percentage
Indications	200	100
Formulation	200	100
Country of origin	200	100
Name and address of the manufacturer	200	100
Active ingredient	196	98
Drug dose	186	93
Storage conditions	186	93
Mechanism of action	147	73.5
Directions for use	125	62.5
Inactive ingredients	106	53
Pharmacokinetic information	106	53
Date of last revision	79	39.5
Duration of treatment	68	34
Container package description	57	28.5
Shelf life	32	16
Provision of full information on request	28	14
Sources of information/reference	4	2
Local language translation	2	1
Retail price of the drug	0	0

**Table 2:** Presence of information on medication package inserts about modifying solid dosage forms (*n* = 146).

Variables	Frequency	Percentage
Instructions to convert tablets or capsules into liquid forms	7	4.8
Possibility of crushing and mixing with food or beverages	6	4.1
Possibility of tablet splitting^*∗*^	10	8.7

^*∗*^ *n* = 115.

**Table 3:** Presence of safety-related information on medication package inserts (*n* = 200).

Variables	Frequency	Percentage
Adverse drug reactions	191	95.5
Contraindications	189	94.5
Drug-drug interactions	160	80.0
Drug-food interactions	42	21.0
Pregnancy considerations	154	77.0
Lactation considerations	148	74.0
Pediatric considerations	141	70.5
Geriatric considerations	75	37.5
Special warning and precautions^*∗*^	99	49.5
Abuse/dependence property	7	3.5
Effect on the ability to drive and operate machines	69	34.5
Overdose and management	139	69.5
Maximum dose	54	27.0
Missing dose	52	26.0
Handling and disposal	45	22.5

^*∗*^Example: renal, hepatic, cardiac, or nutritional insufficiencies that require either increased or reduced dosage.

## Data Availability

The data used to generate this result are available from the corresponding author upon reasonable request.

## Abbreviations

EFDA: Ethiopian Food and Drug Authority
FMHACA: Food, Medicine, and Health Care Administration and Control Authority of Ethiopia
MPIs: Medication package inserts.
